# The importance of epithelial-mesenchymal transition and autophagy in cancer drug resistance

**DOI:** 10.20517/cdr.2019.75

**Published:** 2020-03-19

**Authors:** Charlotte Hill, Yihua Wang

**Affiliations:** ^1^School of Biological Sciences, Faculty of Environmental and Life Sciences, University of Southampton, Southampton SO17 1BJ, UK.; ^2^Institute for Life Sciences, University of Southampton, Southampton SO17 1BJ, UK.

**Keywords:** Epithelial-mesenchymal transition, autophagy, cancer drug resistance, metastasis, therapy

## Abstract

Epithelial-mesenchymal transition (EMT) and autophagy are both known to play important roles in the development of cancer. Subsequently, these processes are now being utilised as targets for therapy. Cancer is globally one of the leading causes of death, and, despite many advances in treatment options, patients still face many challenges. Drug resistance in cancer-therapy is a large problem, and both EMT and autophagy have been shown to contribute. However, given the context-dependent role of these processes and the complexity of the interactions between them, elucidating how they both act alone and interact is important. In this review, we provide insight into the current landscape of the interactions of autophagy and EMT in the context of malignancy, and how this ultimately may affect drug resistance in cancer therapy.

## Epithelial-mesenchymal transition

**Figure 1:**
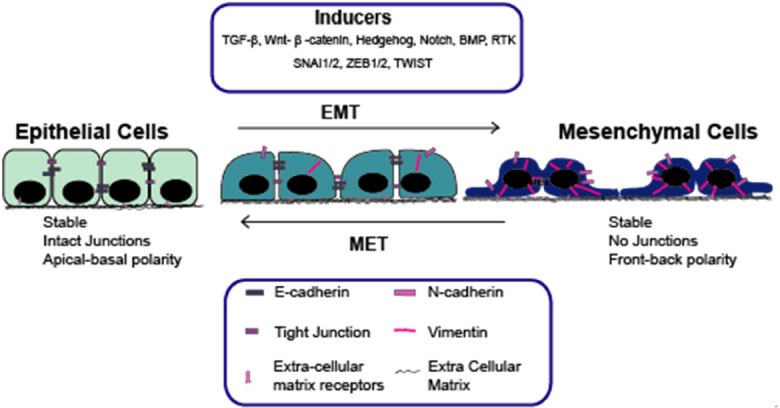
The role of EMT and MET in malignancy, with key biological features and EMT-inducers highlighted. EMT: epithelial-mesenchymal transition; MET: mesenchymal-epithelial transition

Epithelial-mesenchymal transition (EMT) is an important biological process, which is critical in developmental biology and wound healing, but has also been implicated in fibrosis and malignancy^[1-4]^. It is a reversible biological process associated with loss of cell polarity and cadherin-mediated cell adhesion in epithelial cells. These cells transition to mesenchymal cells and, in turn, gain migratory and invasive abilities^[5]^. EMT is mediated through a number of signalling pathways, including transforming growth Factor-beta (TGF-β), Wnt-β-catenin, Hedgehog (Hh), Notch, Bone Morphogenetic Protein and receptor tyrosine kinases^[6]^. Signalling pathways in turn mediate EMT specific transcription factors (EMT-TFs) such as Zinc finger E-box-binding homeobox 1/2 (ZEB1/2), Snail Family Transcriptional Repressor 1/2 (SNAIL1/2) and Twist, which subsequently act to repress the expression of target genes, including E-cadherin. Loss of E-cadherin is considered a key step in EMT^[6-9]^. In the context of malignancy, EMT can result in cells metastasising from primary tumour sites, which has been associated with a worse prognosis [Figure 1].

## Autophagy

**Figure 2:**
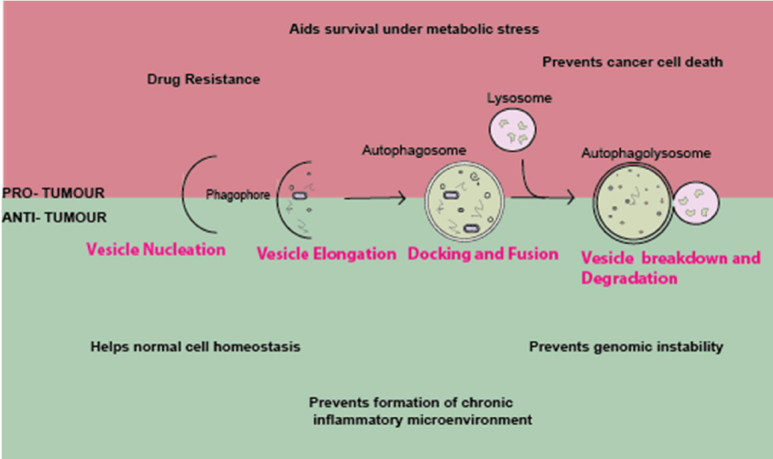
The role of autophagy in cancer: the formation of a double-membrane to engulf material to be degraded by the lysosome by autophagy. The role of autophagy in cancer is complex and has both a pro- and anti-tumour effects. Further discussion in review

Autophagy is an evolutionarily-conserved biological process where long-lived proteins and damaged organelles are degraded by the lysosome^[10,11]^. There are two types of autophagy: general and selective autophagy. In general autophagy, part of the cytoplasm is engulfed, which is delivered to the lysosome and this is degraded. (Macro) autophagy is where a double-membraned vesicle is formed, which captures material in the cytoplasm to be degraded, whereas selective autophagy specifically targets cargo to be degraded^[12-15]^. Autophagy has also been proposed to have roles in a number of diseases^[16]^, such as fibrosis^[17-19]^, neurodegeneration^[20]^ and cancer^[21,22]^ [Figure 2].

The role of autophagy in malignancy is complicated, with conflicting reports on its role in different contexts^[23-27]^. It is thought that autophagy largely aids in tumour suppression in early tumorigenesis, whereas it can promote tumour progression and cancer-cell survival in the later stages. It is understood that autophagy is able to prevent the formation of tumours by maintaining stability in normal cells. During early stages, autophagy protects normal cells from transforming by preventing genomic instability, and thus preventing formation of an inflammatory microenvironment. In comparison, in the later stages, autophagy helps survival of cancerous cells undergoing a number of cellular stresses such as metabolic stress and prevents cell death by anoikis^[28,29]^.

Increased autophagy has been associated with cancer as a mechanism to aid survival and resist treatment^[30]^, with tumours being shown to require autophagy for survival^[31,32]^. As such, autophagy inhibitors have been utilised both alone and in combination with traditional therapy. Several studies demonstrated that autophagy inhibition is able to sensitise cancer cells to further treatment^[33-35]^.

## EMT and autophagy: a complex relationship in malignancy

The signalling pathways of both EMT and autophagy are complex and can be induced in a number of ways; it is therefore unsurprising that there is some interaction between these two pathways^[36,37]^. Numerous studies in different contexts have demonstrated interactions between autophagy and EMT, although it does appear this is both context- and tissue-dependent [Table 1]. Studies have shown that manipulation of autophagy can promote EMT, invasion and metastasis^[21,27,38-40]^; these have been demonstrated in a wide variety of tissues/cell lines including pancreatic, breast, colorectal, melanoma and gastric. In total, 1400 tumours from 20 different types of cancers were analysed for LC3B, an autophagy marker, and it was found that increased expression was associated with metastasis and invasion^[27]^. Autophagy inhibition in rat sarcoma-mutant cancer cells was demonstrated to induce EMT by triggering NF-κB by p62/SQSTM1^[21]^. Similarly, p62/SQSTM1 is important for stabilising Twist1, preventing its degradation^[40]^. In gastric cancer cells, autophagy inhibition promotes EMT and alters the metabolic phenotype of cells, and this is dependent on ROS-NF-κB-HIF-1α^[38]^. In colon cancer cells, Beclin-1 has been shown to be associated with EMT and invasive behaviours; loss of Beclin-1 was able to reverse this phenotype^[41]^. As described above, autophagy has a dual role: in pancreatic ductal adenocarcinoma cells, TGF-β1 induced autophagy in SMAD4-positive cells and inhibited migration by reducing nuclear translocation of SMAD family member 4 (SMAD4), whereas, in SMAD4-negative cells, migration was increased through mitogen-activated protein kinase/extracellular signal-regulated kinase (ERK)^[42]^.

**Table 1:** Autophagy and EMT: the dual role in cancer. Autophagy has been described to have both pro- and anti-tumour effects; some of the recent works in a variety of tissue types where the dual role of autophagy in malignancy has been demonstrated are highlighted

Autophagy	Role on EMT	Cell/Tissue type	Study
**Promotes EMT**			
Inhibition of autophagy	Promotes metastasis through induction of ROS	Gastric cells	[38]
Increased LC3B expression	Associated with metastasis	Breast cancer, melanoma and 18 other cancers	[27]
Autophagy inhibition (ATG KD, histology)	Promotes EMT and invasion	Colorectal cancer, pancreatic cancer (in RAS-mutated cells)	[21]
Autophagy induced by TGF-β	Promotes EMT	NSCL	[39]
Autophagy inhibited (ATG KD)	Promotes EMT via Twist	MEFs, keratinocytes, melanoma cells	[40]
Autophagy induced by TGF-β	Inhibited proliferation, increased migration by MAPK/ERK activation	Pancreatic cancer (SMAD4-neg)	[42]
Inhibition by BECN1	Prevents EMT	Colorectal cancer	[41]
**Prevents EMT**			
Autophagy inhibition	Prevents metastasis	Hepatocellular carcinoma	[43]
Activated autophagy	Prevents EMT by Snail and Twist	Breast	[44]
Autophagy induced by TGF-β	Inhibited migration, promoted proliferation	Pancreatic cancer (SMAD4-pos)	[42]
Autophagy induced by Danusertib (pan-inhibiter of Aurora kinases)	EMT inhibited, potentially via PI3K/Akt/mTOR	Ovarian carcinoma	[45]
Increased autophagy by overexpression of FAT4	Prevents EMT. Regulatory effects of FAT4 on autophagy and EMT are partially by PI3K-AKT	Colorectal cancer	[46]
Increased autophagy	Protects cells from anoikis, promoting luminal filling in early carcinoma	Breast cancer	[47]

EMT: epithelial-mesenchymal transition; ROS: reactive oxygen species; NSCL: non-Small cell lung cancer; TGF-β: Transforming growth factor beta; MAPK: mitogen-activated protein kinase; ERK: extracellular signal-regulated kinase; MEFs: mouse embryonic fibroblasts; RAS: rat sarcoma; ATG: autophagy-related gene; KD: knockdown; PI3Ks: phosphoinositide 3-kinases; AKT: AKT serine/threonine kinase; mTOR: mechanistic target of rapamycin kinase; BECN: Beclin-1; FAT4: FAT atypical cadherin 4

Manipulation of autophagy has also been demonstrated to prevent an EMT-like phenotype and associated metastasis/invasion in a number of cancer cell lines and tissues, including breast, colorectal, pancreatic and ovarian cancers^[42-48]^. In hepatocellular carcinoma, autophagy inhibition was not shown to induce EMT and had no effect on migration or invasion^[43]^. Death effector domain-containing DNA-binding protein attenuates EMT by interacting with Beclin-1 (BECN) and PIK3C3 and activating autophagy^[44]^. In ovarian carcinoma, danusertib induced autophagy, which resulted in suppression of EMT and arrest of G2/M phase, and this may be in part due to P13K/Akt/mTOR signalling^[45]^. Similarly, FAT4 has been shown to regulate activity of phosphoinositide 3-kinases (PI3K) to induce autophagy and inhibit EMT^[49]^.

Given the complicated role of autophagy in malignancy and how several clinical trials are now utilising autophagy inhibitors as treatments for cancer (http://www.cancer.gov/clinicaltrials), the wider-reaching implications of these drugs need to be further investigated.

## Drug resistance: where does EMT come into play?

Drug resistance is a well-known concept where diseases become unaffected by pharmaceutical treatment, which has been studied in a variety of disease models. Two types of drug resistance have been described: acquired and *de novo*^[50]^. Initially, many cancers can be treated with “conventional” therapies such as chemotherapy; however, as the biochemical and tumour environments adapt overtime, sometimes cancer cells become resistant to these treatments. This resistance can be due to many factors, not limited to: drug efflux, metabolism, changes in drug target, DNA damage repair, cell death inhibition and EMT^[51]^.

The link between EMT and drug resistance in cancer was proposed in the 1990s^[52]^ and, subsequently, it has been reported that drug resistance in different cancers is associated with EMT, including lung^[53]^, pancreatic^[54,55]^, bladder^[56]^ and breast cancers^[57,58]^. Activation of several signalling pathways known to induce EMT, such as TGF-β, Wnt, Hh and Notch^[59-62]^, has also been demonstrated to induce cancer drug. Some of the specific mechanisms have begun to be elucidated, but, due to the large variety of drugs, tissue types and signalling pathways involved, it is a complex process, as summarised in Table 2.

**Table 2:** EMT signalling pathways and EMT-TFs: contributions to drug resistance. Several recent studies describe EMT pathways and transcription factors which have been demonstrated to be involved in drug resistance in cancer

		Mechanism of resistance	Tissue type	Study
**Signalling pathway**				
TGF-β		Upregulation of TGFβ	Colon cancer cells	[59]
		-	Triple negative breast cancer	[63]
		-	Squamous cell carcinoma stem cells	[64]
		-	Breast cancer cells (HMLER)	[65]
		Regulating the expression of PDK4	Colorectal cancer	[66]
Wnt		Trastuzumab resistance associated with Wnt3 overexpression activates Wnt/β-catenin which transactivates EGFR	HER2-over expressing breast cancer	[60]
		Resistance to platinum-based chemotherapies. DACT1 demonstrated to be a negative regulator in EOC, inhibiting Wnt signalling and cis-platinum resistance through regulation of autophagy	Type I epithelial ovarian cancer (EOC)	[67]
		NANOGP8 is main regulator. It is closely related to EMT and the Wnt pathway, and correlates with migration, invasion and chemo resistance in gastric cancer	Gastric cancer cells	[68]
Hh		Hh pathway activated in EGFR-WT and EGFR-MT lung cancer	NSCLC	[61]
		Hh pathway activation, EGFR and EPHB3 crosstalk through Hh-STAT3. However, loss of Hh may result in cells being more EGFR-dependent	Colorectal cancer	[69]
Notch		Activation of notch signalling	Pancreatic cancer	[62]
**EMT-TF**				
TWIST	-		Colorectal carcinoma	[70]
	TWIST upregulation		Nasopharyngeal carcinoma	[71]
	Activated Twist mediates P-glycoprotein expression		Bladder cancer	[72]
	-		Breast cells	[73]
Snail1/2	-		Ovarian adenocarcinoma	[74]
	-		HGSOC	[75]
	-		Oral squamous cell carcinoma	[76]
	ABC transporters are overexpressed in cancer and can remove cytotoxic drugs by ATP-dependent efflux. EMT-TF such as TWIST, SNAIL and FOXC2 have been demonstrated to increased levels of ABC transporters, which are directly related to drug resistance		Breast	[77,78]
ZEB1	ZEB1-miR200 feedback loop. ROBO1, OLIG2, CD133 and MGMT identified as novel ZEB1 targets		Glioblastoma	[79]
	Increased IL-1β increases ZEB1 and was associated with increased resistance		Colon cancer	[80]
ZEB2	Loss of FBXW7		Colorectal cancer	[81]

PDK4: pyruvate dehydrogenase kinase 4; DACT1: dapper1 antagonist of catenin 1; EOC: epithelial ovarian cancer; EMT: epithelial-mesenchymal transition; EMT-TF: EMT specific transcription factor; NSCLC: non-small cell lung cancer; Hh: hedgehog; HGSOC: high grade serous ovarian cancer; Wnt: Wingless/Int1; EGFR: epidermal growth factor receptor; WT: wild type; MT: mutant; ABC: ATP-binding cassette; EPHB3: EPH Receptor B3; STAT3: signal transducer and activator of transcription 3; FOXC2: forkhead box C2; ZEB: zinc finger E-box binding homeobox; ROBO1: roundabout guidance receptor 1; OLIG2: oligodendrocyte transcription factor 2; MGMT: O-6-methylguanine-DNA methyltransferase; IL-1β: interleukin 1 beta; FBXW7: F-box and WD repeat domain containing 7

TGF-β signalling has been implicated in different tissues including colorectal, breast and squamous cell carcinoma stem cells^[59,63-66]^, although mechanistically its involvement in drug resistance has been varied. Some studies demonstrated a role for metabolism, showing that TGF-β regulated 5-Fluorouracil (5-FU) resistance in colorectal cancer (CRC) through the regulation of pyruvate dehydrogenase kinase 4^[66]^. In squamous cell carcinoma, TGF-β transcriptionally activates p21, which stabilises NRF2, enhancing glutathione metabolism and reducing the effectiveness of therapies^[64]^. Conversely, downregulation of Smad4 was demonstrated to increase sensitivy in doxorubicin (Dox) resistant colon cancer, which had been shown to be via TGF-β^[59]^. In triple negative breast cancer, TGF-β was shown to be critical in epirubicin resistance by regulating EMT and apoptosis^[63]^. Long-term TGF-β treatment has also been associated with anti-cancer drug resistance^[65]^.

Several other EMT-inducing pathways have also been directly linked to drug resistance in cancer. Wnt has been demonstrated to cause drug resistance in HER2-overexpressing breast cancer, Type-1 epithelial ovarian cancer (EOC) and gastric cancer^[60,67,68]^. In HER2-overexpressing breast cancer cells, it is considered that Wnt3 overexpression may activate Wnt/β-catenin transactivating EGFR, which can lead to a partial-EMT that could be important in understanding trastuzumab resistance in these cells^[60]^. In EOCs, Dapper1 Antagonist of Catenin1 (DACT1) has been shown to negatively regulate Wnt signalling and regulate cis-platinum resistance through regulating autophagy. EOC cells transfected with a lentivirus carrying full-length DACT1 had increased levels of autophagy and were more sensitive to cisplatin^[67]^. In gastric cancer, NANOGP8 overexpression leads to anti-oxaliplatin (L-OHP) resistance. It upregulates EMT markers and increases β-catenin accumulation in the nucleus and strengthens Wnt signalling^[68]^. Activation of the Hh pathway has also been linked to drug resistance in both non-small cell lung cancer with resistance to EGFR-TKIs^[61]^ and in CRC with resistance to cetuximab^[69]^. Finally, the Notch pathway has been implicated in drug resistance in pancreatic cancer. Both Notch-2 and its ligand Jagged-1 are upregulated in gemcitabine-resistant cells and knockdown of Notch resulted in partial reversal of EMT characteristics^[62]^.

Numerous studies in a variety of tissue types have also found EMT-TFs, namely SNAIL1/2, ZEB1/2 and TWIST, to directly confer drug-resistance in cancer^[70-81]^, as summarised in Table 2. Upregulation of these transcription factors alone can be sufficient to confer drug resistance^[71,75]^. ZEB1 is highly expressed in glioblastoma cells, where a ZEB1-miR200 feedback loop connects this with a number of downstream targets (ROBO1, c-MYB and MGMT), and increased levels of this EMT-TF are associated with both drug resistance and reduced survival^[79]^. In CRC, the FBXW7-ZEB2 axis has been demonstrated to control a number of important EMT associated characteristics as well as drug resistance. ZEB2 knockdown was able to reverse the EMT phenotype induced by loss of FBXW7, a tumour suppressor^[81]^. Similarly, overexpression^[70]^ or upregulation^[71,73]^ of TWIST has resulted in chemoresistance in cancer cells; mechanistically, in bladder cancer, this has been shown to be through the upregulation of P-Glycoprotein^[72]^. Several EMT-TFs including TWIST, SNAIL and FOXC2 have been shown to increase levels of ABC transporters. These are overexpressed in cancer and can remove cytotoxic drugs, and therefore increased levels confer drug resistance^[77,78]^. In cisplatin-resistant cell lines, both morphological and phenotypic hallmarks of EMT were identified; gene expression profiling identified several EMT-TFs, including Snail1/2, which were further validated as key players in drug resistance^[74]^. These EMT mechanisms have been demonstrated in a wide-variety of cell lines/tissues including colon, breast, ovarian, gastric and glioblastoma cells, and with a number of different drugs, suggesting a significant issue.

## Autophagy and drug resistance

Autophagy has been implicated in drug resistance in malignancy; chemotherapeutic agents have been shown to be limited in their capacity. They were shown to induce protective-autophagy, and, subsequently, cancer cells became chemoresistant. Cisplatin, a commonly used platinum compound for the treatment of a number of cancers, including ovarian cancer, induces autophagy via ERK and this confers drug resistance in these cancer cells^[82]^. Further, inhibiting autophagy sensitised cancer cells to cisplatin-treatment^[83,84]^, with similar results also found in lung cancer^[85]^. In oesophageal cancer, cisplatin induced autophagy through the class III PI3K pathway and, when cisplatin was used together with autophagy inhibitor 3-Methyladenine, it augmented the effect of the treatment compared to cisplatin alone^[86]^.

Another example of this is 5-FU, which acts by inhibiting DNA synthesis^[87]^, although its ability is ultimately limited as it induces autophagy in cancer cells, which leads to chemoresistance. Several autophagy-related genes have been linked to multi-drug resistance in colorectal carcinoma^[88]^. Blocking autophagy was able to sensitise cancer cells to 5-FU-mediated death^[89,90]^. c-Jun N-terminal kinases (JNK) activation and phosphorylation of Bcl-2 have been demonstrated as key components in 5-FU-induced autophagy in colon cancer^[89]^, where 5-FU-induced autophagy protects cancer cells^[87]^. Similar findings have been shown in gallbladder carcinoma, where 5-FU also induced autophagy, and inhibition of autophagy with chloroquine was able to kill cancer cells^[91]^. Similar findings have been demonstrated in a range of other cancers, including estrogen receptor-positive breast cancer where autophagy inhibition can re-sensitise breast cancer cells to tamoxifen^[92]^. In prostate cancer, high levels of nitrogen permease regulator-like 2, a tumour suppressor candidate gene, can cause resistance to Everolimus by enhancing autophagy via mTOR^[93]^.

Apoptosis and autophagy are closely linked processes and often involved in crosstalk, and it is thought that drug-induced autophagy can protect cancer cells from apoptosis. In breast cancer cells, treatment with Epirubicin induced autophagy in MCF-7 cells and this protected them from drug-induced apoptosis. In drug-resistant MCF-7 cells, autophagy inhibition was able to re-sensitise cells to treatment^[94]^. Finally, three common chemotherapeutics used in the treatment of osteosarcoma induced upregulation of HSP90AA1, which was shown to be a regulator of autophagy via PI3K/Akt/mTOR and apoptosis via JNK/p38^[95]^. Understanding the crosstalk of these pathways in the context of drug resistance will be critical in the development of new therapies.

Given that autophagy and EMT appear to have a complex relationship in malignancy, and that EMT has been demonstrated to contribute to drug resistance, a greater understanding of these relationships is key. New therapeutic strategies are being developed to try to target drug-resistance and targeting autophagy using inhibitors is one of the methods proposed, which was able to sensitise cells to chemotherapy^[96-103]^. Anti-cancer drugs have increasingly been utilised in combination with autophagy inhibitors. When cisplatin was used in combination with autophagy inhibition, this increased cytotoxicity in cells^[48,104]^ Similarly, the effects of 5-FU are augmented in colon cancer when treated with autophagy inhibitor hydroxychloroquine^[105]^.

## Conclusions and future directions

It is clear the underlying mechanisms in cancer drug resistance are multifaceted with several complex, interacting signalling pathways and processes contributing to resistance. These mechanisms are often highly specific, depending on tissue type and stage of disease. Although understanding the implications of these drugs alone on drug resistance is being better elucidated, understanding how these processes interact and the effect this may have on treatment is limited. In many cancers, autophagy inhibitors are being utilised with traditional therapies that can increase cytotoxicity of the drugs. Furthermore, anti-cancer drugs can become resistant through an upregulation of autophagy. However, autophagy inhibition in malignancy has been associated with EMT. Clinically, EMT, in addition to EMT-inducers and EMT-TF, has been linked to cancer-drug resistance. To best optimise treatment, it seems therapies need to be combined, targeted and tissue-specific.

## Declarations

### Copyright

© The Author(s) 2020.
